# Scanning Electron Microscopy and Energy-Dispersive X-Ray Microanalysis of Set CEM Cement after Application of Different Bleaching Agents 

**DOI:** 10.22037/iej.2017.37

**Published:** 2017

**Authors:** Mohammad Samiei, Maryam Janani, Amin Vahdati, Yalda Alemzadeh, Mahmoud Bahari

**Affiliations:** a *Department of Endodontics, Dental School, Tabriz University of Medical Science, Tabriz, Iran; *; b * Dentist, Private Practice, Tabriz, East Azerbaijan, Iran; *; c *Department of Operative Dentistry, Dental School, Tabriz University of Medical Sciences, Tabriz, Iran*

**Keywords:** Bleaching Agents, Calcium-Enriched Mixture, Energy-Dispersive X-Ray Microanalysis, Scanning Electron Microscopy

## Abstract

**Introduction:**

The present study evaluated the element distribution in completely set calcium-enriched mixture (CEM) cement after application of 35% carbamide peroxide, 40% hydrogen peroxide and sodium perborate as commercial bleaching agents using an energy-dispersive x-ray microanalysis (EDX) system. The surface structure was also observed using the scanning electron microscope (SEM).

**Methods and Materials:**

Twenty completely set CEM cement samples, measuring 4×4 mm^2^, were prepared in the present *in vitro* study and randomly divided into 4 groups based on the preparation technique as follows: the control group; 35% carbamide peroxide group in contact for 30-60 min for 4 times; 40% hydrogen peroxide group with contact time of 15-20 min for 3 times; and sodium perborate group, where the powder and liquid were mixed and placed on CEM cement surface 4 times. Data were analyzed at a significance level of 0.05 through the one Way ANOVA and Tukey’s post hoc tests.

**Results:**

EDX showed similar element distribution of oxygen, sodium, calcium and carbon in CEM cement with the use of carbamide peroxide and hydroxide peroxide; however, the distribution of silicon was different (*P*<0.05). In addition, these bleaching agents resulted in significantly higher levels of oxygen and carbon (*P*<0.05) and a lower level of calcium (*P*<0.05) compared to the control group. SEM of the control group showed plate-like and globular structure. Sodium perborate was similar to control group due to its weak oxidizing properties. Globular structures and numerous woodpecker holes were observed on the even surface on the carbamide peroxide group.

**Conclusion:**

The mean elemental distribution of completely set CEM cement was different when exposed to sodium perborate, carbamide peroxide and hydrogen peroxide.

## Introduction

Bleaching of non-vital teeth is an inexpensive and conservative alternative for restorative techniques [1]. However, the root canal orifice(s) of endodontically treated teeth should be sealed with various materials, including glass-ionomer, mineral trioxide aggregate (MTA), *etc*, in order to prevent the penetration of bleaching agents into periodontal tissues *via* the dentinal tubules so that external cervical root resorption can be prevented [2, 3]. It is obvious that under such conditions the bleaching agents can change the physical and chemical properties of the root canal sealing agent [4]. *In vitro* animal models have shown that hydrogen peroxide used in bleaching procedures can exert deleterious effects on the physical properties such as color, microhardness and surface roughness of materials used to seal the root canal orifices [4]. On the other hand, in the majority of cases the orifice plug material remains in place permanently and thus, they should preserve their physical properties [4]. 

Different materials have been used for this purpose to seal the root canal, which are minimally affected by bleaching agents. Of these materials, zinc oxide-eugenol, IRM, glass-ionomer, bonding materials and MTA can be mentioned [4].

Calcium-enriched mixture (CEM) cement has rapidly become an important material in endodontics due to its good physical properties [5, 6]. Capabilities such as a proper seal in the presence of blood and moisture, biocompatibility and hard tissue induction potential are important properties of materials such as MTA and CEM cement, which are useful for purposes such as filling the root-end cavities, root canal treatment of immature teeth and pulp capping [7]. Moradi *et al.* [6] evaluated CEM cement and MTA in relation to their apical seal and reported that CEM cement can provide a proper seal against body fluids and microorganisms in a manner similar to MTA. CEM cement has exhibited better physical properties, including flow and film thickness, in comparison with MTA; in addition, it has a shorter setting time [8] Several studies have investigated the properties of this material [9-13] 

It is believed that CEM cement might be used to seal the root canal orifices during tooth bleaching procedures [14]. Maleknejad *et al.* [15] evaluated the ultra-structural changes in dentin after exposure to different bleaching agents (45% carbamide peroxide, 35% hydrogen peroxide, sodium perborate plus 35% hydrogen peroxide, and sodium perborate plus water) using an electron microscope and reported no significant differences between the experimental subgroups in relation to calcium and phosphorus. However, the weight percentages of calcium, phosphorus and sulfur in dentin were significantly lower in bleached group compared to the control group, with significantly higher weight percentage of potassium compared to the control group [15].

The surface changes and physical properties of MTA after the application of various chemicals and physiological solutions has been evaluated [16]. Furthermore, the structural changes of MTA after the application of hydrogen peroxide (H_2_O_2_) solution as acidic condition was investigated using scanning electron microscopy (SEM) and the energy-dispersive x-ray microanalysis (EDX) system [17].

To date no studies have evaluated the applicability of CEM cement as orifice sealing material during bleaching procedures. The interaction between CEM cement and other dental materials is important. Therefore, the aim of the present *in vitro* study was to evaluate the mean elemental distribution and surface topography of CEM cement after application of conventional bleaching agents (35% carbamide peroxide, 40% hydrogen peroxide and sodium perborate) on completely set CEM cement.

The null hypothesis was that structural deterioration, together with change in the elemental level, occurs after the application of bleaching gel to CEM cement.

## Materials and Methods

CEM cement powder and liquid (BioniqueDent, Tehran, Iran) were mixed according to the manufacturer instructions and the mixed cement was placed in 25 silicon tubes (4 mm in length and 4 mm in diameter) and maintained in a container at room temperature in relative humidity. 

The samples were completely set with no surface cracks. After complete setting which was determined by hardness, samples were randomly divided into 4 groups; 3 groups (*n*=5) were exposed to bleaching agents, consisting of 40% carbamide peroxide (WHITE smile, Birkenau, Germany), sodium perborate and 35% hydrogen peroxide (WHITE smile, Birkenau, Germany) and one group served as the control group.

The following clinical standard bleaching methods recommended by manufacturers were used in this study. Carbamide peroxide was placed in 1 mm-thick layers on CEM cement and kept for 30-60 min. After that, the surface was cleaned with tap water for 1 min and dried with air. This procedure was repeated four times (according to the manufacturer’s instructions). 

Sodium perborate was mixed with distilled water and placed in 3 mm-thick layers on CEM cement and kept for 72 h. Then, the surface was cleaned with tap water for 1 min and dried with air. This procedure was repeated four times within three days.

Hydrogen peroxide was placed with 1-2 mm thickness on the surface of CEM cement and kept for 15-20 min. After that, the surface was cleaned with air for 1 min and this procedure was repeated three times. 

After the last use of bleaching, the surface of all samples was cleaned with tap water for 1 min and dried with air. The samples were mounted on aluminum holders with adhesive carbon tape and observed using an operating microscope. Thereafter in order to make samples conductive, they were coated with carbon using a vacuum evaporator (JEE-4C, JEOL Ltd., Japan). Then the samples were evaluated under SEM (MIRA3 FEG-SEM) connected to EDX (EMAX7000 Type S; Czech) SEM observations were carried out under ×50, ×250 and ×500 magnifications with the use of secondary electron imaging technique at kVp=15 and a distance of 15 mm. In the present study, the variables evaluated consisted of mean elements of oxygen, sodium, calcium, silicon and carbon after the application of three different bleaching agents.

Data were analyzed using the Statistical Package for Social Science (SPSS, version 18.0, SPSS, Chicago, IL, USA) through the one way ANOVA and Means-Test-Tukey’s tests. The level of significance was defined as 0.05.

## Results

***EDX***

Based on the results, carbamide peroxide and hydrogen peroxide resulted in similar element distribution of oxygen, sodium, calcium and carbon; however, they caused changes in the element distribution of silicon (*P*=0.005). In addition, these bleaching agents resulted in significantly higher oxygen and carbon (*P*<0.05) and lower calcium levels (*P*=0.001) compared to the control group. The calcium levels were similar to those of the control group. Sodium perborate resulted in the highest levels of oxygen and sodium (*P*<0.05) and in the lowest levels of calcium, carbon and silicon (*P*=0.005) (Tables 1and 2).

***SEM***

**Figure 1:**
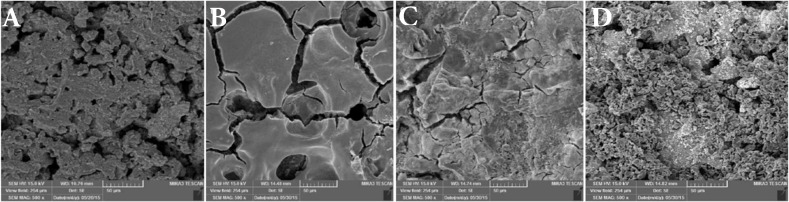
*A)* SEM of the control group; *B)* SEM of the surface of CEM cement in the carbamide peroxide group; *C)* SEM of CEM cement surface cement in the sodium perborate group; *D)* SEM of the surface of CEM cement in the hydrogen peroxide group

In the control group the surface had plate-like and globular structure (Figure 1A). Globular structures and numerous woodpecker holes (around 38 m in diameter) were observed on the even surface on the carbamide peroxide group (Figure 1B).

Sodium perborate was similar to control group due to the weak oxidizing properties (Figure 1C).

In hydrogen peroxide the surface was not only globular or plate-like but also some cracks were formed (Figure 1D). 

## Discussion

Bleaching agents result in changes in the elements and also organic and inorganic compounds in both restorative materials and tooth enamel. Changes in the inorganic compounds show that they are affected by the bleaching agents and changes in elements such as sulfur show that these inorganic ingredients are also affected groups compared to the control group, which might be attributed to the oxidative properties of bleaching agents and the release of hydrogen peroxide [17]. In addition, the amount of oxygen in the sodium perborate group was significantly higher. The data were obtained using the SEM and EDX and showed significantly higher concentrations of oxygen in all the bleached than other groups. In a study by Mozayeni *et al. *[17] a combination of sodium perborate and 30% H_2_O_2_ exhibited the highest bleaching efficacy. It should be pointed out that the high concentration of oxygen in the sodium perborate group can be attributed to measurement errors resulting from the chalky state and the high adhesive property of this bleaching agent to CEM cement so that it was not completely removed from the CEM cement surface. 

**Table 1:** Comparison of mean element levels of completely set CEM cement in different bleaching groups

**Group (N=5) **	**Mean (SD)**				
	**Oxygen**	**Sodium**	**Calcium**	**Silicon**	**Carbon**
**Control **	35.25 (1.75)	4.03 (0.46)	28.83 (1.46)	6.07 (0.29)	12.02 (0.55)
**Sodium perborate **	64.87 (3.75)	17.86 (3.34)	4.98 (1.27)	2.36 (1.72)	8.84 (2.13)
**Carbamide peroxide **	44.72 (3.02)	2.86 (0.58)	18.15 (5.63)	2.54 (1.21)	29.63 (6.89)
**Hydrogen peroxide **	47.09 (4.05)	1.53 (1.22)	13.38 (9.19)	15.42 (9.04)	20.75 (4.32)
***P*** -value*	0.004	0.005	0.000	0.006	0.000

**Table 2:** Two-by-two comparison of bleaching groups in relation to the mean element levels of completely set CEM cement

		***P*** **-value**				
		**Oxygen**	**Sodium**	**Calcium**	**Silicon**	**Carbon**
**Control**	**Sodium perborate**	0.000	0.002	0.000	0.262	0.381
**Control**	**Carbamide peroxide**	0.043	0.729	0.021	0.318	0.000
**Control**	**Hydrogen peroxide**	0.014	0.465	0.002	0.017	0.014
**Sodium perborate**	**Carbamide peroxide**	0.001	0.001	0.010	0.789	0.000
**Sodium perborate**	**Hydrogen peroxide**	0.002	0.000	0.079	0.002	0.004
**Carbamide peroxide**	**Hydrogen peroxide**	0.567	0.679	0.234	0.001	0.009

The results of the present study showed significantly lower amounts of calcium in all bleached groups compared to the control group, which is consistent with the results of previous studies [15, 18-21]. Since CEM cement contains different calcium compounds, a higher concentration of calcium in the control group indicated that the bleaching agents dissolved and removed calcium from the surface of CEM cement due to their oxidative properties. Carbamide peroxide and hydrogen peroxide contain a relatively similar amount of calcium in their structure. Moreover, Maleknejad *et al.* [15], showed that these two bleaching agents have a similar effect on calcium. 

In the present study, the concentration of calcium in the sodium perborate group was significantly less than carbamide peroxide and hydrogen peroxide group. Given the lower oxidative property of sodium peroxide, this bleaching agent is safe and can be controlled [22, 23]. 

The results of the present study showed no significant differences in the concentration of sodium between the groups bleached with carbamide peroxide and hydrogen peroxide. A higher concentration of sodium on the surface of CEM cement in this group might be attributed to the presence of calcium in the structure of the bleaching agent, which remained on the surface of CEM cement. Therefore, the concentration of sodium in the samples bleached with sodium perborate was higher than the control group, and other bleached groups had sodium concentrations similar to the control group. 

The results of this study revealed that the amount of silicon in the hydrogen peroxide group was significantly higher than other groups, with similar amounts of silicon in the control, sodium perborate and carbamide peroxide groups. Tsujimoto *et al. *[20] showed that hydrogen peroxide resulted in an increase in the concentration of silicon in MTA, which was proportional to the concentration of the bleaching agent. Studies by Turker and Biskin [24] bleaching agents affect the SiO_2_ ingredients of restorative materials.

CEM cement contains calcium silicate crystals (tricalcium silicate, C_3_S and dicalcium silicate, C_2_S) [5]. This material undergoes dissolution of calcium silicate crystals under the influence of bleaching agents. Calcium is dissolved in the bleaching materials (gel, solution, *etc.*) and SiO_2_ remains on the CEM cement surface. A higher concentration of silicon in the hydrogen peroxide group compared to the other study groups was attributed to the strong effect of hydrogen peroxide and dissolution of crystals; in this context, an increase in free radicals results in disintegration of molecular bonds (Tables 1 and 2).

The results of the present study showed that the concentration of carbon in the sodium perborate group was similar to the control group, with significantly higher concentrations in the carbamide peroxide and hydrogen peroxide groups. A higher concentration of carbon in the carbamide peroxide group is due to the presence of this element in the composition of carbamide peroxide (CH_6_N_2_O_3_). Carbamide peroxide disintegrates into urea and hydrogen peroxide in the oral cavity [23]. Therefore, a higher concentration of sodium in this group is due to the residual carbon from carbamide peroxide.

An increase in the concentration of carbon in the hydrogen peroxide group is due to the high oxidative effect of this material and its effect on the dissolution of CEM cement constituents. CEM cement contains calcium carbonate which is disintegrated upon contact with hydrogen peroxide, resulting in an increase in the concentration of carbon on the restorative material [25].

## Conclusion

In conclusion, the present findings suggest that an acidic environment of bleaching agents necessitates the protection of CEM cement lining with an intermediate resin before bleaching procedure.
